# Plateletcrit is predictive of clinical outcome and prognosis for early‐stage breast cancer: A retrospective cohort study based on propensity score matching

**DOI:** 10.1002/cam4.6944

**Published:** 2024-02-01

**Authors:** Xu Zhao, Yilan Yang, Zhe Pan, Weiluo Lv, Xinxin Rao, Xuanyi Wang, Xiaoli Yu

**Affiliations:** ^1^ Department of Radiation Oncology Fudan University Shanghai Cancer Center Shanghai China; ^2^ Department of Oncology, Shanghai Medical College Fudan University Shanghai China; ^3^ Shanghai Clinical Research Center for Radiation Oncology Shanghai China; ^4^ Shanghai Key Laboratory of Radiation Oncology Shanghai China

**Keywords:** blood parameters, disease‐free survival, early‐stage breast cancer, plateletcrit

## Abstract

**Purpose:**

Breast cancer (BC) is diagnosed as the most common cancer in women in 2022 according to the American Cancer Society. It is essential to detect early and treat early. Several studies have shown that some blood parameters have important predictive value for BC. In this study, we aim to explore whether some immune‐associated blood parameters are relevant to disease‐free survival (DFS) in early‐stage BC.

**Methods:**

A single‐center, regression cohort study of 1490 patients with early‐stage BC in Shanghai Cancer Center was conducted from January 2008 to December 2016. The patients were matched according to the ratio of 1:1 based on Propensity Score Matching (PSM). All patients who experienced disease progression were matched successfully. Thus, 58 pairs of subjects were obtained. Matched blood parameters were evaluated by paired samples t‐test or Wilcoxon signed‐rank test. Factors with statistical difference were further evaluated by stratified COX regression model.

**Results:**

Univariate analysis showed differences in platelet‐related parameters (PLT, PCT, and PLR) and NLR between the two matched groups. However, stratified COX regression analysis, which ruled out the confounding effects of multiple factors, found that only PCT had prognostic value in early BC patients at baseline and study endpoint. Meanwhile, platelet‐related parameters (PLT, MPV) and NLR were different in the progressive group by self before and after comparison. However, the multiple‐factor analysis showed that only the NLR had prognostic value. ROC curve analysis indicated that the best sensitivity (65.45%) and specificity (78.18%) were obtained when the baseline PCT was 0.225. The optimal sensitivity (70.91%) and specificity (65.45%) were obtained when the PCT of disease progression was 0.215. The Kaplan–Meier curve was used to calculate the DFS rate based on the critical values of the two groups.

**Conclusions:**

Some blood parameters have value to predict DFS in early‐stage BC patients, especially platelet‐associated parameters.

## 1 BACKGROUND

Based on the latest statistical data by the American Cancer Society in 2022, breast cancer (BC) is widely recognized as the most commonly diagnosed tumor in women, accounting for about one‐third of female tumors. And the incidence of BC has slightly increased since 2004. Due to the rapid proliferation, metastasis and drug resistance property of breast neoplastic cells, BC has become the second largest killer with an estimated mortality rate of 15%.^1^ It is particularly important to detect and treat this malignant tumor early for tumor management. In recent years, many countries have widely carried out the screening programs targeting the early BC. So, the prognosis of this kind of patients has been steadily improved.^2^ There are several factors that are considered as independent prognostic variables of BC traditionally, such as tumor size, lymph node metastasis, clinical stage, pathological grade, and molecular types.^3^ Besides, mounting evidence suggests that inflammatory factors play an important role in the occurrence and recurrence of tumors. Certain subset of breast neoplasms has a rich immune microenvironment.^4^ Immune cells infiltrating in the tumor microenvironment (TME) mainly encompass tumor infiltrating lymphocytes (TILs), dendritic cells (DCs), tumor‐associated macrophage (TAM) and tumor‐associated neutrophil (TAN). These cells are closely related to homologous cells within the blood. The risk of recurrence will decrease by 15%–23% for each 10% increase of TILs.^5^, ^6^, ^7^ In addition, many studies have proved that different immune states link to different tumor prognosis.^8^, ^9^, ^10^ Most published research findings mainly focus on the lymphocyte‐related blood parameters (LRBP), including absolute lymphocyte counts (ALC), neutrophil‐to‐lymphocyte ratio (NLR) and platelet‐to‐lymphocyte ratio (PLR). Various reports have found that ALC, NLR, and PLR are exactly correlated with the prognosis of BC.^11^, ^12^, ^13^ However, few studies have laid emphasis on the platelet‐related parameters. As an important factor, platelets are proved to be indispensable in tumors recently. Platelets are the main components of thrombosis and hemostasis in the human body. Moreover, they also play a vital part in many other physiological and pathophysiological processes, like inflammation, angiogenesis and cancer progression.^14^ With profound understanding of tumors, researchers illustrated that platelets in the TME also have some functions. When platelets circulate into the TME, platelets could bind to tumor‐related molecules. Consequently, the growth of the primary tumor is supported and the secondary metastases are established. These platelets are known as tumor‐educated platelets (TEP).^15^

Since 2015, both preclinical and clinical researches have been conducted to explore whether platelet‐related parameters could predict the prognosis of BC. In a 4T1 BC mouse model, researchers observed the changes in primary tumor weight, lung metastasis and blood cell parameters after implanting the BC cells to mice. Importantly, platelet‐related parameters of 4T1 mice, such as platelet (PLT), plateletcrit (PCT), mean platelet volume (MPV) and platelet distribution width (PDW), were significantly increased (*p* < 0.05). It can be inferred that platelet parameters are probably related to primary tumor growth and lung metastasis.^16^ What's more, one clinical study showed that pre‐treatment MPV was associated with poor pathologic features and prognosis in patients with invasive BC.^17^

By collecting clinical data of 1490 patients with early BC in Shanghai Cancer Center from January 2008 to December 2016, we conducted a retrospective study to find out if there is a relationship between blood routine test indicators and the prognosis of patients with early BC. Special attention has been paid to platelet‐related indicators, which may have clinical predictive value.

## 2 METHODS

### 2.1 Study population and design

This retrospective study reviewed a total of 1490 patients with early‐stage BC who achieved standardized treatments in Shanghai Cancer Center from January 2008 to December 2016. In this study, we defined early‐stage BC as ductal carcinoma in situ (DCIS) and ductal carcinoma in situ with microinvasion (DCISM). All patients received surgery, including total mastectomy or breast‐conserving surgery. Whether achieved lymph node treatment, adjuvant chemotherapy, adjuvant radiotherapy (RT), adjuvant hormonal therapy or Herceptin treatment is based on patient's condition and doctor's recommendations. Patients were excluded if they have the previous malignancy history, neoadjuvant therapy, follow‐up less than 6 months, concurrent invasive carcinoma, Paget's disease and incomplete immunohistochemistry (IHC) of ER and PR. Patients' clinical data including general condition (age), postoperative pathological conditions (tumor size, nuclear grade, microinvasion, necrosis, lymph node metastasis) and important immunohistochemical conditions related to BC (ER status, PR status, HER2 status) were recorded. There were 58 patients whose disease has progressed in the study cohort. Although the baseline parameters of all patients have no statistical difference, not every patient did blood tests when they had return visits. So, we used Propensity Score Matching (PSM) by age, nuclear grade, micro‐infiltration status, necrosis status, lymph node status, ER status, PR status and HER2 status to match these 58 patients with the others who had completed blood routine tests for the purpose to minimize the differences of these confounders.

Tumor size was classified according to the RTOG 9804 trial.^18^ The grade of tumor nuclear was assessed on the basis of the Nottingham grading system. DCIS with micro‐infiltration meant cancer cells extend beyond the basilar membrane no more than 1 mm of the greatest dimension into the connective tissues based on the 2019 version 5 of “WHO BC Histological Classification”.^19^ The judgment of nuclear necrosis was still based on the above reference. Lymph node metastasis was determined by postoperative pathology. The histopathological features of ER, PR, and HER2 were assessed by immunohistochemistry. ER or PR was considered positive if ≥5% tumor cells had staining.^20^ According to the ASCO/CaP guidelines, HER2‐positive status was defined as HER2 IHC 3+ or HER2 gene amplification+ by fluorescence in situ hybridization (FISH). All data in this study were approved by the institutional review board of Shanghai Cancer Center.

### 2.2 Blood samples and data collection

We used the blood tests taken at the first visit of the patients to our hospital as the baseline. The routine blood records measured at the time of the follow‐up when recurrence or metastasis was detected were used as the study endpoint. And the blood tests used as controls in the matched group were taken at similar length of follow‐up time. Patients' complete peripheral blood parameters (including absolute lymphocyte, neutrophil and platelet counts, mean platelet volume) were collected at baseline and study endpoint. These parameters could be used to calculate PCT, NLR and PLR. PCT was provided by multiplying platelet counts by mean platelet volume. NLR was calculated by the ratio between the absolute neutrophil counts and the ALC. Likewise, PLR was calculated by dividing the absolute platelet numbers by the absolute lymphocyte numbers.

### 2.3 Follow‐up and study endpoint

The objective of this investigation was to find out if any blood parameters have value to predict the prognosis of the early‐stage BC patients. Follow‐up visits of all patients were scheduled every 3 months within the first 2 years, biannually in the next 3 years, and annually thereafter. The study endpoint was disease‐free survival (DFS), which was considered as survival without distant metastasis, local regional recurrence or contralateral BC. Local regional recurrence included disease recurrence of local breast or chest wall or the ipsilateral lymph node basins.

### 2.4 Statistical analysis

IBM SPSS Statistics 26 was used to analyze statistics and do PSM. For categorical variables, the data was described as frequency. If continuous variables conformed to the normal distribution, the data were expressed as mean ± standard deviation. Otherwise, data were expressed in quartiles (median [quartile 25%, 75%]). The baseline characteristics was compared by independent samples *t*‐test, Mann–Whitney *U*‐test or Pearson's chi‐squared test. The matched samples were analyzed by paired samples t‐test, wilcoxon signed‐rank test or McNemar's test. Multivariate analyses of all matched parameters were evaluated by stratified COX regression model. The cutoff optimizations were calculated by using ROC curve analysis, which were selected by the biggest Youden index. Meanwhile, the predictive value of categorical variables was assessed. The Kaplan–Meier method was used to distinguish between the survival analyses of two matched groups. And the significant differences were identified by log‐rank test. All statistical tests used were two‐sided and *p*‐values <0.05 were considered to be statistically significant in this study.

## 3 RESULTS

### 3.1 Patient characteristics

All patient baseline characteristics were shown in Table 1. Before PSM, the median age of the disease‐progressive group was 50.47 ± 11.97 years old and the two groups were about the same. 66.8% of patients in the non‐disease‐progressive group had primary breast tumors smaller than 2.5 cm, while the proportion of the disease‐progressive group was about 67.0%. According to the histopathologic findings, there were 304 cases with DCISM in the non‐disease‐progressive group. And in this group, there were 2.4% of patients with nuclear necrosis, 86.0% of patients with negative lymph node, 77.0% of patients with positive estrogen receptor, 70% of patients with positive progesterone receptor and 24.5% of patients with positive HER2 status. Some of the 1490 patients we included underwent breast‐conserving surgery and some underwent total mastectomy. Depending on the clinical symptoms and imaging manifestations of the patient's axilla, some had axillary sentinel lymph node biopsy (SLNB), some had axillary lymph node dissection (ALND) while some had their axilla left untreated. We also took whether they had received adjuvant therapy (postoperative chemotherapy, postoperative radiotherapy, postoperative targeted therapy, and postoperative endocrine therapy) in consideration. These variables showed no significant difference among the two groups by statistical analysis.

**TABLE 1:** Baseline characteristics before and after Propensity Score Matching of Study Cohorts.

Variables	Before PSM			After PSM		
	Non‐disease progression (*n* = 1432)	Disease progression (*n* = 58)	*p* Value	Matched group (*n* = 58)	Research group (*n* = 58)	*p* Value
Age	50.42 ± 10.75	50.47 ± 11.97	0.976	51.14 ± 8.99	50.47 ± 11.87	0.71
Tumor size, cm						
≤2.5	957	40	0.944	41	40	0.779
>2.5	292	11		10	11	
Unknown	183	7		7	7	
Nuclear grade						
Low	301	10	0.940	9	10	0.938
Low to intermedia	160	5		8	5	
Intermedia	456	20		20	20	
Intermedia to high	133	5		5	5	
High	297	14		12	14	
Unknow	85	4		4	4	
Microinfiltration						
No	1128	49	0.295	49	49	1
Yes	304	9		9	9	
Necrosis						
No	1398	55	0.362	57	55	0.5
Yes	34	3		1	3	
pN						
Negative	1231	46	0.063	51	46	0.132
Positive	11	2		2	2	
Unknow	190	10		5	10	
ER						
Negative	330	16	0.422	16	16	1
Positive	1102	42		42	42	
PR						
Negative	429	18	0.861	18	18	1
Positive	1003	40		40	40	
HER2						
Negative	636	25	0.081	27	25	0.392
Positive	351	21		19	21	
Unknow	445	12		12	12	
LN						
Non‐operation	222	13	0.053	8	13	0.253
SLNB	850	31		27	31	
ALND	340	11		20	11	
SLNB+ALND	20	3		3	3	
Breast						
Conserving surgery	317	15	0.504	12	15	0.510
Total mastectomy	1115	43		46	43	
Radiotherapy						
Yes	245	16	0.082	9	16	0.114
No	1163	42		49	42	
Unknow	24	0		0	0	
Chemotherapy						
Yes	218	9	0.623	14	9	0.352
No	1191	49		44	49	
Unknow	23	0		0	0	
Target therapy						
Yes	23	1	0.831	2	1	1
No	1400	57		56	57	
Unknow	9	0		0	0	
Endocrine therapy						
Yes	801	32	0.689	32	32	1
No	614	26		26	26	
Unknow	17	0		0	0	

Abbreviations: ALND, axillary lymph node dissection; ER, estrogen receptor; HER2, Human Epidermal Growth Factor Receptor 2; LN, lymph node; PR, progesterone receptor; PSM, Propensity Score Matching; SLNB, sentinel lymph node biopsy.

However, some of the 1490 patients lacked blood tests. So, we matched patients with similar characteristics and focused on the blood parameters of these selected.

### 3.2 Comparison of blood parameters between two matched group

As shown in Table 2, platelet‐related parameters did differ between the two groups at baseline, with absolute platelet count and plateletcrit significantly higher in the disease progression group than in the matched group (P < 0.001). And PLR was also different (P = 0.041). In addition, the comparison of blood parameters between the two groups at the endpoint (Table 3) showed that the three platelet‐related parameters mentioned above differed as well. Moreover, the NLR was higher in patients with a worse prognosis (P = 0.035).

**TABLE 2:** Baseline blood test characteristics between the matched group.

Variables	Matched group	Research group	*p* Value
LYM (10^9^/L)	1.943 ± 0.0916	1.948 ± 0.560	0.960
GRAN (10^9^/L)	3.1 [2.5–3.825]	3.25 [2.675–4.025]	0.823
PLT (10^9^/L)	201.57 ± 43.10	232.67 ± 49.25	**<0.001** ***
MPV (fL)	9.940 ± 1.357	10.262 ± 1.14	0.123
PCT	0.197 ± 0.040	0.237 ± 0.049	**<0.001** ***
NLR	1.836 ± 0.847	1.850 ± 0.688	0.918
PLR	110.315 [86.97–132.805]	116.61 [101.015–143.1975]	**0.041** *

Bold values indicates statistically different.

Abbreviations: LYM, absolute lymphocyte count; MPV, mean platelet volume; NLR, neutrophil‐to‐lymphocyte ratio; PCT, Plateletcrit; PLR, platelet‐to‐lymphocyte ratio; PLT, absolute platelet count. Bold values: statistically different.

*** *p* < 0.001.

* *p* < 0.05.

**TABLE 3:** Endpoint blood test characteristics between the matched group.

Variables	Matched group	Research group	*p* Value
LYM (10^9^/L)	1.8[1.4–2.3]	1.7[1.4–2.1]	0.612
GRAN (10^9^/L)	3.3[2.5–4.1]	3.7[2.8–4.4]	0.121
PLT (10^9^/L)	199.13 ± 39.86	222.51 ± 48.13	**0.007** *
MPV (fL)	10.218 ± 0.940	10.607 ± 1.177	0.075
PCT	0.202 ± 0.037	0.233 ± 0.041	**<0.001** ***
NLR	1.75 [1.43–2.41]	2.13 [1.44–3.00]	**0.035** *
PLR	104.29 [90.56–133.57]	123.57 [105.26–150.63]	**0.033** *

Bold values indicates statistically different.

Abbreviations: LYM, absolute lymphocyte count; MPV, mean platelet volume; NLR, neutrophil‐to‐lymphocyte ratio; PCT, plateletcrit; PLR, platelet‐to‐lymphocyte ratio; PLT, absolute platelet count.

*** *p* < 0.001.

* *p* < 0.05.

In order to avoid confounding effects among factors, we further used stratified COX regression to conduct multi‐factor analysis of blood indexes concerned in this study. It was found that only PCT at both baseline and endpoint varied significantly between the matched two groups (P < 0.001).

### 3.3 Comparison of blood parameters in the disease progression group

In view of the differences in the blood parameters between the two matched group, we would like to further analyze whether there were changes in the blood parameters of the research group between the baseline timepoint and the disease progression timepoint (Table 4). Univariate analysis showed that the baseline absolute platelet count was higher (P = 0.023) but the MPV was lower (P = 0.019). Interestingly, the NLR rose as the disease progressed (P = 0.004).

**TABLE 4:** Blood test characteristics of the research group between baseline timepoint and disease progression timepoint.

Variables	Baseline	Endpoint	*p* Value
LYM (10^9^/L)	1.966 ± 0.567	1.816 ± 0.705	0.087
GRAN (10^9^/L)	3.480 ± 1.218	3.773 ± 1.156	0.066
PLT (10^9^/L)	233.636 ± 49.572	222.510 ± 48.130	**0.023** *
MPV (fL)	10.251 ± 1.1651	10.607 ± 1.1769	**0.019** *
PCT	0.237 ± 0.0497	0.233 ± 0.0410	0.443
NLR	1.71[1.345–1.710]	2.13[1.44–3.00]	**0.004** *
PLR	116.61 [101.015–143.1975]	123.57 [105.26–150.63]	0.224

Bold values indicates statistically different.

Abbreviations: LYM, absolute lymphocyte count; MPV, mean platelet volume; NLR, neutrophil‐to‐lymphocyte ratio; PCT, plateletcrit; PLR, platelet‐to‐lymphocyte ratio; PLT, absolute platelet count.

* *p* < 0.05.

By further stratified COX regression, a statistically significant difference only in NLR was elucidated (P = 0.011).

### 3.4 Prognostic significance of some blood parameters

**FIGURE 1:**
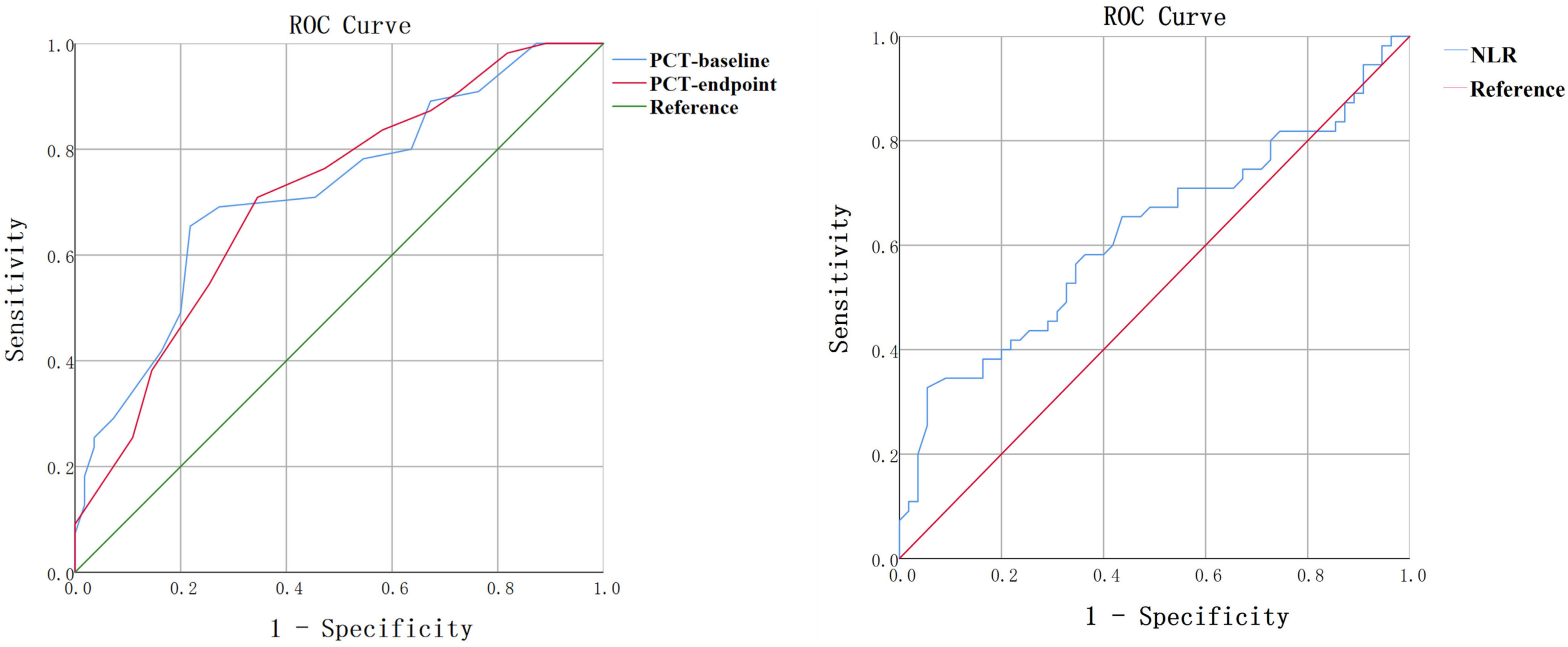
ROC curves of predictive indicators (plateletcrit and neutrophil‐to‐lymphocyte ratio).

ROC curves (Table 5) were used to analyze the above variables with statistical differences. The results revealed that the area under curve (AUC) of the baseline PCT was 0.722. And the optimum sensitivity (65.45%) and specificity (78.18%) were obtained when the baseline PCT was 0.225. The AUC of the study endpoint PCT was 0.712, and the optimal sensitivity (70.91%) and specificity (65.45%) were obtained when this parameter value was 0.215. Using the same method, the NLR had the best threshold when this value is 2.72. The sensitivity was 32.7% and the specificity was 94.55%. The illustration is shown in Figure 1.

**TABLE 5:** ROC curve results and the cutoff values of predictive indicators.

Variables	AUC	Sensitivity	Specificity	Youden Index	Cutoff	95% CI	*Z* Value
Baseline PCT	0.722	65.45%	78.18%	0.4363	0.225	0.627–0.817	**<0.001** ***
Endpoint PCT	0.712	70.91%	65.45%	0.3636	0.215	0.617–0.808	**<0.001** ***
NLR	0.621	32.7%	94.55%	0.2725	2.72	0.515–0.727	**0.028** *

Bold values indicates statistically different.

Abbreviations: AUC, Area Under Curve; NLR, neutrophil‐to‐lymphocyte ratio; PCT, plateletcrit.

*** *p* < 0.001.

* *p* < 0.05.

### 3.5 Plateletcrit has independent prognostic value

**FIGURE 2:**
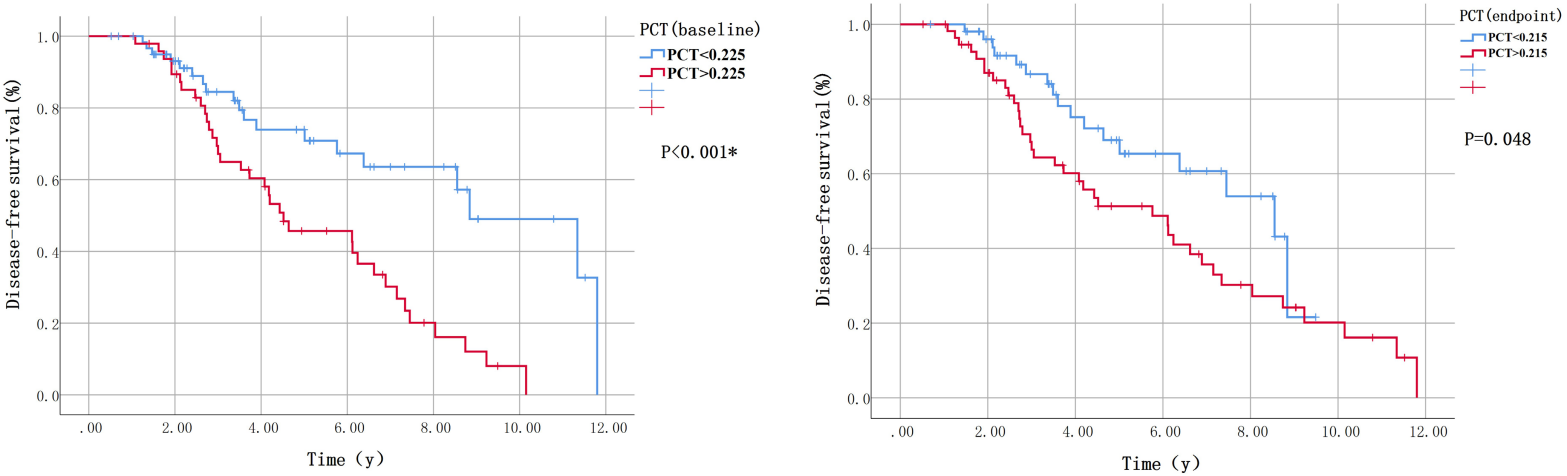
Kaplan–Meier curves illustrating disease‐free survival (DFS) of high Plateletcrit group and low Plateletcrit group.

We took 0.225 as a cutoff value of the baseline PCT and divided all matched patients into high baseline PCT group and low baseline PCT group for Kaplan–Meier curve analysis. As a result, the baseline PCT did have an impact on the prognosis of early BC patients. Similarly, all matched patients were divided into a high PCT group and a low PCT group when at the time of our study endpoint with a cutoff value of 0.215, Kaplan–Meier curve analysis showed comparable results (Figure 2).

## 4 DISCUSSION

Recent reports have confirmed that platelets play a role in cancer metastasis and angiogenesis. For example, platelets enable to promote tumor angiogenesis by producing several biological factors such as vascular endothelial growth factor (VEGF), serotonin and endostatin.^21^ In addition, platelets are the main producer of TGFβ1, which can lead to cancer invasion by launching epithelial‐mesenchymal transition (EMT) of cancer cells.^22^ Through platelet surface molecule P‐selectin, platelets can adhere to cancer cells and bind to CD24 ligand on the natural killer (NK) cells, thus avoiding NK cell‐mediated lethal cytotoxicity and driving tumor growth.^23^ The nitric oxide (NO) secreted by platelets is a bioactive compound that regulates angiogenesis and immune response.^24^ Activated platelets also release lysophosphatidic acid (LPA) and platelet factor 4 (PF4), which are crucial in the growth and invasion of tumor cells.^25^, ^26^ Hence, Platelets are of great importance in cancer progression and metastasis.

Researches have already validated that PLT is a potential prognostic marker for colorectal cancer and endometrial cancer.^27^, ^28^, ^29^ According to many recent studies, platelet‐related indicators are in connection with cancer prognosis as well, such as PLT, PCT, MPV, PDW, and PLR. PCT is obtained by multiplying PLT and MPV, reflecting the platelet mass per unit volume.^30^ PCT varies between patients with different types of cancers and healthy people.^31^, ^32^ Ma et al. found that epithelial ovarian cancer patients had higher PCT compared with healthy controls.^31^ However, another study showed that the PCT of the lung cancer patient group was lower compared to the control group. And the PCT value in the lung cancer patients with distant metastasis was higher than that in the lung cancer patients without distant metastasis.^32^ Therefore, PCT can be used as a diagnostic and prognostic indicator for some cancers. But the exploration of PCT in BC is still very limited. A clinical trial of advanced BC showed that lower baseline PCT levels in patients were associated with better overall survival.^33^

For all we know, this study was the first to show that PCT was significantly correlated with disease progression in early‐stage BC patients. And it was an important factor affecting the prognosis. In this retrospective study of 1490 subjects, we evaluated the predictive value of PCT in patients with early‐stage BC based on PSM. We found that PCT was related to poor prognosis in patients. Furthermore, all matched patients were regrouped according to the ROC curve using the cutoff value. As were shown in figures, the DFS of patients in the low PCT group was significantly longer than that in the high PCT group. Besides, increased NLR level during posttreatment follow‐up visits may also indicate disease progression in patients with early BC.

## 5 CONCLUSION

The prognostic markers of BC are especially important. ER, PR, and HER2 are factors we are familiar with.^34^ More novel markers are being explored for the past few years. Human homologs of seven in absentia (SIAH) expressed in the residual tumor was proved to be a new biomarker to forecast the tumor relapse by leveraging the activation or inactivation of EGFR/K‐RAS/SIAH pathway recently.^35^ CircRNAs are also one of the research spotlights. It was illustrated that One circRNA called circEZH2 could promote the oncogenesis of BC by the FUS/circEZH2/KLF5/CXCR4 positive feedback loop.^36^ These markers may potentially be used as targets in oncologic areas for biological therapies. In our study, we primarily paid attention to the blood test. Blood tests are more convenient to monitor and more accessible than tissue biopsy. For patients, these indicators are easy to record and the measurement cost is low. Nevertheless, the absolute counts of these cells are easily affected by various physiological conditions. The relative indexes (NLR, PLR, and PCT) are relatively stable.^37^ Our research suggested that elevated PCT and poor prognosis in patients with early BC were necessarily interrelated. Also, NLR may predict disease progression if it has a tendency to increase during follow‐up. Combined with the result of the previous analysis, it might be inferred that the NLR index increases and is higher than normal if the disease progresses. This is consistent with the results of many previous literature reports on the prediction of BC prognosis by using blood routine test.^38^, ^39^ The lymphocytes, neutrophils and platelets are closely related to components contained in the TME discovered, which might be the theoretical basis of our analysis.

However, not every patient had routine blood tests during the period of all treatments and follow‐up. So, the total number of patients included in the study was 1490 but only 58 pairs of patients' blood routine indexes were finally used for analysis. The sample size was small and all came from the same institution. Though this retrospective study has some inherent limitations, the potential predictive value of PCT and NLR for early‐stage BC can't be neglected.

## AUTHOR CONTRIBUTIONS

**Xu Zhao:** Methodology (equal); writing – original draft (equal). **Yilan Yang:** Data curation (equal); resources (equal). **Zhe Pan:** Investigation (equal). **Weiluo Lv:** Investigation (equal); methodology (equal). **Xinxin Rao:** Conceptualization (equal); data curation (equal). **Xuanyi Wang:** Methodology (equal); software (equal). **Xiaoli Yu:** Funding acquisition (equal); validation (equal); writing – review and editing (equal).

## FUNDING INFORMATION

This work was supported by the National Natural Science Foundation of China 81972846.

## CONFLICT OF INTEREST STATEMENT

The authors declare that they have no competing interests.

## ETHICS STATEMENT

The whole procedures in our study involving human data were performed based on the 1964 Helsinki Declaration and the ethical standards of the national research committee. All 1490 patients signed the written informed consent during hospitalization for surgery. They were fully informed and consented to researchers using information during treatment in Shanghai Cancer Center for subsequent scientific research, including all relevant clinical data. Ethics Committee Board of Fudan University Shanghai Cancer Center approved the study.

## Data Availability

The data that support the findings of this study are available from the corresponding author with the permission of Fudan University Shanghai Cancer Center, upon reasonable request.
